# The Efficacy and Safety of Afatinib in Non-Small Cell Lung Cancer Patients with Brain Metastasis: A Meta-Analysis

**DOI:** 10.1155/2023/5493725

**Published:** 2023-03-02

**Authors:** Hui Jin, Ping Liang, Juan Hou, Bin Li, Ping Wang, Xin He

**Affiliations:** ^1^Department of Medical Oncology, The Fourth Hospital of Hebei Medical University, Shijiazhuang, Hebei 050000, China; ^2^Department of Pharmacy, The Fourth Hospital of Hebei Medical University, Shijiazhuang, Hebei 050000, China; ^3^Department of Respiratory, The Fourth Hospital of Hebei Medical University, Shijiazhuang, Hebei 050000, China

## Abstract

**Aim:**

The aim of this study is to evaluate the efficacy and safety of afatinib in the treatment of non-small cell lung cancer (NSCLC) patients with brain metastasis based on meta-analysis.

**Methods:**

Related literatures were searched in the following databases: EMbase, PubMed, China Knowledge Network (CNKI), Wanfang, Weipu, Google Scholar, the China Biomedical Literature Service System, and other databases. Clinical trials and observational studies that met the requirements were selected for meta-analysis using Revman 5.3. The hazard ratio (HR) was used as an indicator of the impact of afatinib.

**Results:**

A total of 142 related literatures were acquired, but after screening, five literatures were selected for data extraction. The following indices were compared: the progression-free survival (PFS), overall survival (OS), and common adverse reactions (ARs) of grade 3 and above. A total of 448 patients with brain metastases were included and were divided into two groups: the control group (no afatinib treatment, with chemotherapy alone and the first-generation EGFR-TKIs) and the afatinib group. The results showed that afatinib could improve PFS (HR: 0.58, 95% CI: 0.39–0.85, *P* < 0.05) and ORR (OR = 2.86, 95% CI: 1.45–2.57, *P* < 0.05), but had no benefit on OS (HR: 1.13, 95% CI: 0.15–8.75, *P* > 0.05) and DCR (OR = 2.87, 95% CI: 0.97–8.48, *P* > 0.05). For the safety of afatinib, the incidence of grade-3-and-above ARs was low (HR: 0.01, 95% CI: 0.00–0.02, *P* < 0.05).

**Conclusion:**

Afatinib improves the survival of NSCLC patients with brain metastases and shows satisfactory safety.

## 1. Introduction

The incidence of advanced non-small cell lung cancer (NSCLC) with brain metastases is 20–40% and increases to 44–63% in EGFR-positive patients [1, 2]. The traditional treatment for NSCLC patients with brain metastasis is whole-brain radiotherapy (WBRT). However, after receiving WBRT, cognitive dysfunction will lead to a decrease in the quality of life. With the application of a tyrosine kinase inhibitor (TKI), the survival of NSCLC patients with brain metastasis has been significantly prolonged, with a median overall survival (OS) of about 14.8 months [3–5].

The epidermal growth factor receptor (EGFR) signaling pathway plays an important role in carcinoma [6]. In recent years, the use of EGFR-TKIs as a targeted therapy for NSCLC patients with brain metastases has been widely practiced. Afatinib, as a second-generation EGFR-TKI, is a powerful and irreversible dual inhibitor of EGFR and HER2 tyrosine kinases, with a longer inhibition time and elevated efficacy [7–10]. The NCCN guidelines have listed EGFR-TKIs as the first-line treatment for advanced NSCLC with EGFR mutations [7, 11]. However, afatinib has not been recommended as the first-line agent for NSCLC patients with brain metastases. EGFR-TKIs have a small molecular weight, good fat solubility, and a great ability to penetrate the blood-brain barrier (BBB). It has been shown that afatinib can easily penetrate the blood brain barrier to control the brain metastasis of EGFR-mutant NSCLC [9]. However, there is a lack of multicenter clinical evidence currently. Therefore, a meta-analysis was performed in this study by including different studies on the treatment of NSCLC patients with brain metastasis with afatinib, and the results will provide a reference for promoting the clinical application of afatinib.

## 2. Materials and Methods

### 2.1. Inclusion and Exclusion Criteria

PROSPERO registration number for this meta-analysis is 373151. According to the PICOS principle, the inclusion criteria were as follows: (1) Aged at least 18 years, advanced recurrent or metastatic NSCLC (confirmed by histology or cytology) with EGFR mutation, the brain metastasis was confirmed by CT or MRI. (2) Intervention measures included at least two groups: control and afatinib (single afatinib or afatinib combined with other treatments), (3) with the following data: primary outcome indicators including progression-free survival (PFS), overall survival (OS), and hazard ratio (HR), and secondary outcome indicators including treatment-related deaths and AEs (Grade 3 or 4, according to the National Cancer Institute's common toxicity standards). (4) Research types: RCT, non-RCT, observational studies (cohort studies, case-control studies, and cross-sectional studies); the language should be either Chinese or English. Animal experiments, mechanistic studies, repeated publications, case reports, and reviews were excluded.

### 2.2. Search Strategy

The following databases were searched: CNKI, Wanfang, PUBMED, EMBASE, CBM, OVID, Cochrane library, ClinicalTrials, and OpenGrey. The latest date was May 31, 2021. The search terms were “Afatinib” or “Gilotrif,” “non-small cell lung cancer” or “lung cancer,” “brain metastases” or “CNS.” For each acquired paper, we also reviewed its references. Our analysis was in accordance with the “Cochrane Intervention System Evaluation Manual” and the PRISMA statement.

### 2.3. Literature Screening

The inclusion criteria for literature were as follows: (1) A study that included patients with NSCLC and brain metastases; (2) There are clear result data verifying the safety and/or efficacy of afatinib; (3) If multiple articles were published based on the same original data, only the latest and most complete articles were used.

### 2.4. Data Extraction

Two investigators independently conducted the search process and evaluated the included articles, and any inconsistencies were discussed until a consensus was reached. For each article, we collected the first author, publication year, number of registrations, number of patients, treatment plan, and demographic factors (such as age and histological type). Besides, a treatment for patients who had not received systemic treatment (e.g., chemotherapy) before was defined as the first-line treatment, and a treatment for patients who had received platinum-based chemotherapy was defined as the second-line treatment.

### 2.5. Statistical Analysis

The Revman 5.3.0 software was used for efficacy meta-analysis focusing on PFS, OS, and hazard ratio (HR). First, the heterogeneity was evaluated, if there was significant heterogeneity (*I*^2^ > 50%, *P* < 0.1), we analyzed the reasons (such as clinical heterogeneity), then subgroup analysis was conducted to eliminate the heterogeneity. If the heterogeneity was not due to clinical heterogeneity but statistical heterogeneity, the random effects model was used for pooled analysis. If there was no heterogeneity (*I*^2^ ≤ 50%, *P* ≥ 0.1), the fixed effects model was used for pooled analysis. If the sample size was sufficient, a funnel plot analysis was performed to observe whether there was a publication bias.

## 3. Results

### 3.1. Search Results

**Figure 1:**
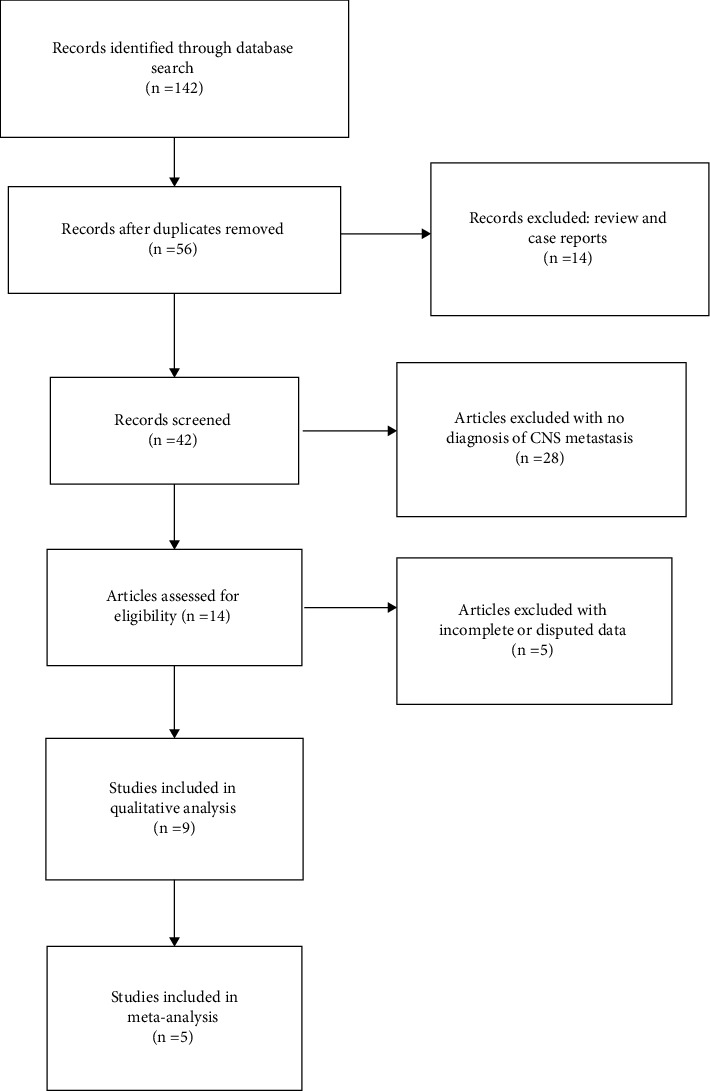
Flow diagram of the study selection process.

We acquired 142 articles from all available databases. After reviewing the title and abstract, fifty-six articles were included. After reading the full text and checking the indices, nine articles were included, and finally five articles were screened for meta-analysis [9, 12–15]. The flowchart is shown in Figure 1.

### 3.2. Basic Characteristics of Included Studies

For the nine included studies, they are all cohort studies. All cases included were confirmed NSCLC patients with brain metastases, and afatinib was used in the treatment. The possible factors related to the risk of brain metastasis were recorded, including age, histological characteristics, the dose of afatinib (40 mg), the early-stage treatment for brain metastasis (such as WBRT), and drugs used in the control group (such as platinum, gemcitabine, erlotinib, pemetrexed, etc.). To evaluate the efficacy and safety of afatinib, the median PFS, OS, and HR of each group were extracted (Table 1).

### 3.3. Afatinib Prolongs the PFS of NSCLC Patients with Brain Metastasis

**Figure 2:**
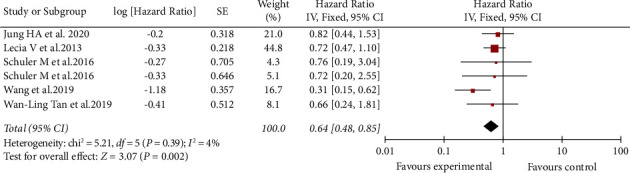
Meta-analysis of PFS of afatinib in brain metastases.

In the included five studies, the total HR of afatinib as the first-line treatment is 0.58 (95% CI: 0.39–0.85), and the inverted funnel figure shows no publication bias (Figure 2). This result proves that afatinib can prolong the PFS of NSCLC patients with brain metastasis.

### 3.4. Afatinib Has No Effects on OS

**Figure 3:**
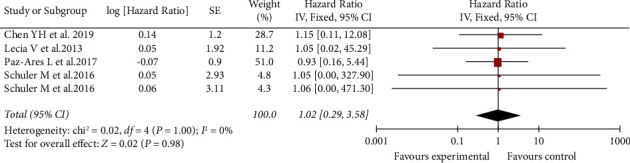
Meta-analysis of OS of afatinib in brain metastases.

In the three included studies, the heterogeneity of OS was extremely low (*I*^2^ = 0%), and its overall HR was 1.13 (95% CI: 0.15–8.75). There is no significant difference in OS between afatinib and control therapies (gemfibrozil, erlotinib, or chemotherapy) (Figure 3).

### 3.5. Afatinib Provides Better Objective Response Rate (ORR) and Disease Control Rate (DCR)

**Figure 4:**
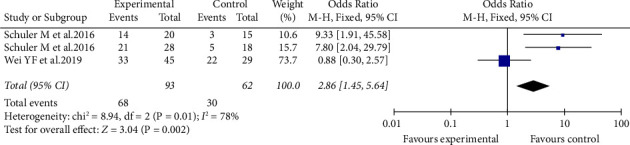
Meta-analysis of ORR of afatinib in brain metastases.

**Figure 5:**
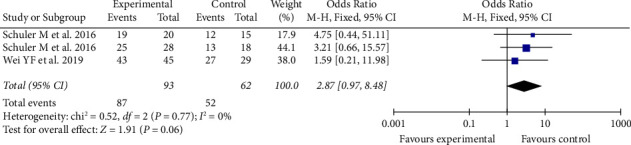
Meta-analysis of DCR of afatinib in brain metastases.

In the three included studies, the results of the ORR were heterogeneous (*I*^2^ > 50%), and the odds ratio (OR) is 2.86 (95% CI: 1.45–2.57). In comparison with chemotherapy, afatinib can provide a better ORR (Figures 4 and 5). Meanwhile, the heterogeneity of the disease response rate (DCR) is extremely low (*I*^2^ = 0%), and the odds ratio (OR) is 2.87 (95% CI: 0.97–8.48).

### 3.6. Adverse Reactions (ARs) of Afatinib in the Treatment of NSCLC with Brain Metastases

**Figure 6:**
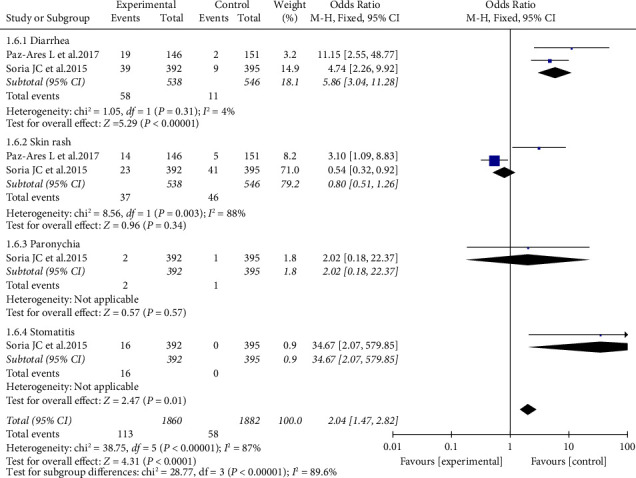
Meta-analysis of overall AR of afatinib in brain metastases.

The common ARs were investigated [19, 20]. Overall, the incidence of ARs is high. The overall OR of ARs is 1.56 (95% CI: 1.44–1.70). Diarrhea, stomatitis, and paronychia were among the most common ones. The overall OR of Grade ≥ 3 ARs is 2.04 (95% CI: 1.47–2.82). Diarrhea was among the most common one (Figure 6).

## 4. Discussion

The incidence and mortality of lung cancer are still remarkably high currently [17]. About 10% of patients have brain metastasis at the time of diagnosis, and about 40% may develop brain metastasis during treatment [16]. At present, the commonly used local treatments for this cohort are: WBRT, stereotactic radiotherapy (SRT), and surgical resection. The local treatments play an important role in combination with systemic therapies (such as chemotherapy, immunotherapy, and EGFR TKI combined therapy).

Afatinib is an oral ErbB-family blocker [8]. Compared with the first-generation EGFR TKI, afatinib binds to EGFR irreversibly and suppresses the carcinogenic signaling pathway. Afatinib can control tumor progression and improve the prognosis of EGFR mutant NSCLC patients with brain metastasis [9]. By reviewing real-world data, this study used first-line chemotherapy or the first-generation EGFR-TKIs as the control group and observed the efficacy and safety of the afatinib group as the first-line therapy. In the afatinib group, the HR of PFS is 0.58 (95% CI: 0.39–0.85), with a reduced risk of progression. The OR of the ORR is 2.86 (95% CI 1.45–2.57) and the OR of the DCR is 2.87 (95% CI 0.97–8.48) in comparison with controls, suggesting that afatinib can provide a better treatment response. This is consistent with the conclusion of the LUX-Lung3/6 trials [9]. In these trials, especially among the brain metastasis cohort, the PFS of the afatinib group was significantly better than the chemotherapy group. In the LUX-Lung 3 trial, the PFS of the afatinib group was 11.1 months, and that of the cisplatin-pemetrexed was 5.4 months. In the LUX-Lung 6 trial, the PFS periods of afatinib and cisplatin-gemcitabine groups were 8.2 months and 4.7 months, respectively. However, we found no benefits of afatinib on OS (HR 1.13, 95% CI: 0.15–8.75). In the LUX-Lung3/6 trails, a negative result of OS was also reported in the total population, but an OS benefit was observed in the EGFR19del subgroup. So far, although afatinib can control the progression of brain metastasis, it does not bring OS benefit versus chemotherapy or the first-generation EGFR-TKIs. In the treatment efficacy assessment, afatinib had a better ORR and DCR versus chemotherapy and the first-generation EGFR-TKI. Overall, this is also consistent with the conclusion of the LUX-Lung3/6 trails. It should be noted that we did not stratify the oral dose of afatinib in our selected studies because a meta-analysis reported no difference between the efficacy of the 30-mg group and the 40-mg groups [21].

The common AEs of afatinib include skin rash, diarrhea, loss of appetite, stomatitis, vomiting, fatigue, and paronychia. Compared with the first-generation EGFR-TKIs, afatinib has a higher incidence of stomatitis, diarrhea, and paronychia among all grades of AEs, and the incidence of rashes is similar. Among grades 3 and above AEs, afatinib is associated with a higher incidence of diarrhea, while the incidences of rash, stomatitis, and paronychia are similar.

At present, it is still disputable whether TKIs in combination with radiotherapy are beneficial for controlling brain metastasis. Radiotherapy may provide clinical benefits for brain metastasis caused by NSCLC with rare EGFR mutations [22]. Early treatment such as WBRT and chemotherapy can increase the permeability of the BBB. Therefore, a pretreatment of radiotherapy and chemotherapy before the use of afatinib may assist afatinib in penetrating the BBB [23]. In the LUX-lung 3/6 trial, among patients who had received WBRT, afatinib can significantly improve PFS and OS in elderly patients [24]. However, there are also controversial conclusions [18]. A retrospective study of NSCLC patients with EGFR-sensitive mutation and brain metastasis found that adding WBRT to EGFR-TKI therapy has no additional survival benefit [25]. Another retrospective study showed that the OS of patients receiving early radiotherapy was similar to that of the delayed radiotherapy group [26]. A meta-analysis also showed that only the EGFR-TKI group (but not the WBRT plus EGFR-TKI group) showed superior intracranial PFS. Therefore, it is still too early to answer this question, and further clinical trials and real-world data are needed.

The limitations of this meta-analysis are as follows. First, the small sample size weakens the reliability of the results. Second, we include several retrospective observational studies, and there might be selection bias in these studies.

In conclusion, this meta-analysis suggests that afatinib improves the survival of NSCLC patients with brain metastases and shows satisfactory safety.

## Figures and Tables

**Table 1:** Main characteristics of each study included.

First author	Year	Study design	Included studies	Treatment status	Number of BM	Age (years)/population	Pre-treatment	Dose (mg)	Quality score
Schuler [9]	2016	Coh	Lux-lung 3 adenocarcinoma	First-line	42	62/Asian	WBRT	40	6
		Coh	Lux-lung 6 adenocarcinoma	First-line	49	54/Asian	WBRT	40	6

Li [6]	2018	Coh	Adenocarcinoma	First-line	28	60/Asian	NA	40	6
Su [16]	2018	Coh	NA	First-line	306	60/Asian	NA	40	6
Tan [15]	2019	Coh	Adenocarcinoma	First-line	42	62/Asian	WBRT	30	6
Wang [13]	2019	Coh	Adenocarcinoma	First-line	24	58/Asia	SRTWBRT	40	6
Chen [14]	2019	Coh	Stage III B or IV adenocarcinoma	First-line	134	65/Asian	Radiotherapy of brain metastases	40	6
Lin [17]	2019	Coh	Adenocarcinoma	First-line	125	60/Asian	NA	40	6
Wei [18]	2019	Coh	Advanced lung adenocarcinoma	First-line	74	60/Asian	NA	40	6
Jung [12]	2020	Coh	Adenocarcinoma	First-line	157	60/Asian	SRTWBRT	40	6

## Data Availability

All data are available upon reasonable request to the corresponding author.
